# Errors in Results and Figure

**DOI:** 10.1001/jamanetworkopen.2025.50945

**Published:** 2025-12-01

In the Original Investigation titled “Facility-Based Uptake of Colorectal Cancer Screening in 45- to 49-Year-Olds After US Guideline Changes,”^1^ published November 4, 2025, there were errors in the Results and Figure. In the first paragraph of the Results, the mean monthly volume increased by 948%, not 955%, and the number of individuals aged 50 to 75 years was 52 348 prechange and 76 503 postchange (not 520 683 prechange and 2 286 703 postchange). In the second paragraph of the Results, Hispanic representation increased by 2.8%, not 7.4%. Finally, the y-axis of the Figure should have started at −200%, not 0%. This article has been corrected.^1^
